# Proteolytic enzyme arbitrated antagonization of helminthiasis by *Cinnamomum cappara* leaf extract in *Pheretima posthuma*

**DOI:** 10.1186/s40816-021-00261-9

**Published:** 2021-02-18

**Authors:** Kayeen Vadakkan, Meena K Cheruvathur, Anu S Chulliparambil, Famy Francis, Anu P Abimannue

**Affiliations:** 1 Department of Biotechnology, St. Mary’s College, 680020 Thrissur, Kerala India; 2 Department of Botany, St. Mary’s College, 680020 Thrissur, Kerala India; 3 Marian Centre for Advanced Research, St. Mary’s College, 680020 Thrissur, Kerala India

**Keywords:** *Cinnamomum cappara*, Lineweaver–Burk plot, *Pheretima posthuma*, Proteolytic action

## Abstract

**Background:**

There have been several studies carried out to irradiate Helminthiasis however very little research have been carried out where in the enzymatic activity of plants are exploited to antagonize infections. Here we are analyzing the anthelmintic activity of *Cinnamomum cappara* leaf extract against *Pheretima posthuma* complimented by proteolytic action.

**Results:**

The fresh leaves of Cinnamomum cappara was collected from local areas of Thrissur during December 2019. Plants were identified and authenticated by morphological and molecular characterization. The enzymatic action was analyzed by plotting Lineweaver–Burk plot which suggested that the extract possess the Km 185.77 μM for casein as substrate and obeyed Michaelis–Menten kinetics with typical hyperbolic relation with enzyme and increasing concentration of substrate. The effect of extract upon study subject was in directly proportional with concentration of antagonist where higher activities were obtained in high concentrations. The anatomical and histological studies suggested that the activity of extract was due to the degradation of muscular bundle of subject that resulted in the leakage of ceolomic fluid.

**Conclusions:**

Cinnamomum cappara leaf extract possessed high degree of protease intervened anthelmintic activity against Pheretima posthuma. As the study subject show immense morphological and physiological resemblance with all other helminthic parasites, this results shall be adopted to further clinical and pharmacological applications.

## Introduction

Helminthiasis has proclaimed itself as one among the most important parasite animal diseases in the planet as it affect more than a 25 % of the total world’s population and effect in significant disease and disability. The increasing amount of contaminated soil catalyses the transmission, and infections with *Ascaris lumbricoides*, *Trichuris trichiura*, and hookworm [1].Worldwide it causes notable economic instability especially in developing countries.The lack of knowledge about proper and adequate management of such parasitic outbreak exacerbate the scenario [2].Helminths can be classified into two major phyla among which first one comprises of the nematodes which include the major intestinal worms those are soil-transmitted helminths and also filarial worms that could cause lymphatic filariasis, onchocerciasis etc. The second category comprises of platyhelminths that embraces the flukes and tapeworms and this group could cause schistosomes, and cysticercosis [3]. Conventionally the usage of chemical parasiticides are preferred to control Helminthiasis, however over usage of such agents could result in depreciation that in turn result in environment and living organisms, It could also give rise to drug resistant parasites [4].

The recognition of the antigenic complexity of parasites has slowdown the vaccine development in the past the growth in systems biology have brought some new velocity to the area, however lack in parallel advances in product and clinical development sciences and the potential for a candidate vaccine to advance into clinical trials remains low. This decelerate the preclinical development, and clinical development of vaccines [5]. The possibilities of employing plant metabolites for the treatment of Helminthiasis can replace the conventional chemical drug mediated treatment. It is assumed that the plant derived natural drugs will have more compatibility and less toxic effects when compared that with its chemical counterparts [6]. Even though there have been several plants have been reported for its successful anthelminthic activity, the activity responsible for the action remains mostly unknown.Current phase of anthelminthic drug development demands novel strategies to overcome existing obstacles encountered [7]. In this study we analyze the effect of proteolytic enzyme depended antagonization of Helminthiasis by *Cinnamomum cappara* leaf extract.

## Materials and Methods

### Collection and identification of plant material

The fresh leaves of *Cinnamomum cappara* was collected from local areas of Thrissur during December 2019. Plants were identified and authenticated by a botanist Dr. Sr. Meena K Cheruvathur, Assistant professor, Department of Botany, St. Mary’s College, Thrissur, Kerala and kept in herbarium under voucher number SMCTSR/CC/2019. Further the identity of the plant was confirmed by the employment of sequencing rbcL gene marker [8]. Amplification of marker gene was done by the employment of universal primer and resulting nucleotide was sequenced. Thus obtained nucleotide sequence was analyzed for its phylogenetic relationship using nucleotide blast available in NCBI (National Centre for Biotechnological Information) home page.

## Extraction of bioactive fraction by decoction method

The plant material was dried in shade at ambient temperature, and made it in to powder by electrical blender. The powdered material was stored in tightly closed glass bottles for further use. The crude aqueous extract of *Cinnamomum cappara* leaves was prepared using the powdered plant material by mixing it with 250 mL of distilled water in a flask which was then boiled for 30 minutes. The mixture was boiled until the volume reduced to 50 ml. Thus obtained crude extract was cooled and later filtered using what man filter paper 1 mm and collected in a reagent bottle. [9].

### Analysis of proteolytic activity by Cinnamomum ***cappara*** leaf extract

The quantitative determination of protease was carried as follows. The reaction mixture was made by the crude extract containing 50 µg protein in it, 0.5 ml of Tris–HCl buffer (50 mM; pH 7.2) and 0.5 ml of casein (2 % w/ v) dissolved in citrate phosphate buffer (50 mM; pH 6.8). The mixture was left to react at 37 °C for 1 h and the reaction was arrested by adding 1 ml of 10 % (w/v) ice - cold TCA. The unreacted casein substrate was cleared away by centrifugation at 5,000 rpm for 10 min, later an aliquot (0.5 ml) of the supernatant was taken and 2.5 ml of the reagent comprising 2.9 % Na2CO3 and 0.3 N NaOH was added and followed by the addition of 0.75 ml of Folin Ciocalteu’s phenol reagent. The samples were incubated at 37 °C for 20 min and the absorbance was taken at 650 nm. A standard curve constructed with tyrosine was utilized to calculate the protease activity [10]. One unit of protease activity (U/ml) was defined as the amount of enzyme that liberates1µmol of tyrosine equivalent per minute under the assay conditions. From the obtained value the specific activity (U/mg) was derived by dividing total activity by total protein content. The total protein content of the leaf extracts was determined by the method of Bradford, (1976) wherein BSA was used as standard to generate the calibration curve in Bradford assay The effect of increasing substrate concentration upon rate of reaction was studied in constant reaction conditions such as pH 8.0 and 37 ℃ where in 20 mg/ml crude plant extract was added to solution. The concentration of substrate varied from 1 to 150 µM in each mixture. A blank was used in each specific substrate concentrations without the enzyme. The Michaelis–Menten constant (Km) value was derived from Lineweaver–Burk plot [11].

### Activity dependent purification of bioactive fraction

Crude Aqueous extract from *Cinnamomum cappara* leaf possessing positive proteolytic activity was subjected for silica gel column chromatography (60–120 mesh) and the bioactive compounds were eluted by the employment of appropriate solvents of varying polarity. Eluted fractions were screened for its activity; finally, positive fraction was concentrated and the lead component was identified by the employment of gas chromatography (Agilent 6890 series) equipped with HP-5MS column mass spectrometer [12] operated at initial column temperature of 30 °C and heated up to 300 °C at 10 °C /5 min. Chromatographic conditions were: flow of 1.0 ml /min of high purity helium as carrier gas in split mode. The identification of the compounds in spectra was done based on retention time and integral area of peaks. The similarity of compounds matched based on NIST library search [13].

### Collection and maintenance of ***Pheretima posthuma***

All the experiments were carried out in Indian adult earthworms (*Pheretima posthuma*) due to its anatomical resemblance with the intestinal roundworm parasites of human beings. *Pheritima posthuma* with the average sze of 5–8 cm length was procured from Kerala Agricultural University, Vellanikkara, Thrissur, Kerala, India. The animals were maintained in moisture soil in the lab supplied with proper aeration [14].

### Experimental design for screening protease enzyme dependent anthelmintic activity

The anthelmintic activity was performed according to the method. On adult Indian earth worm *Pheretima posthuma* as it has anatomical and physiological resemblance with the intestinal round worm parasites of humans. *Pheretima posthuma* was placed in petridish containing 25 ml of test solution of varying concentrations of *Cinnamomum cappara* leaf extract ranging from 0 to 40 mg/ml. Each Petri dish was placed with 6 worms and observed for paralysis or death. Mean time for paralysis was noted when no movement of any sort could be observed, except when the worm was shaken vigorously; the time death of worm (min) was recorded after ascertaining that worms neither moved when shaken nor when given external stimuli [15]. Albendazole (50 mg/ml) was used as the positive control while distilled water was considered as negative control. The observations obtained from the test was compared that with positive and negative control.

### Histological analysis of study subjects

After the incubation period the treated samples were collected and washed with distilled water. After a depuration of 6 h, as recommended by [16]. Earthworms were sequentially dehydrated in graded ethanol concentrations and embedded in paraffin wax with different melting points. Slides with cross sections of earthworms were stained using the hematoxylin–eosin method for light microscope observation, and photographed with an Labomed microscope equipped with a digital color camera Micaps pro HDMI [17].

### Statistical analysis

The data obtained from the study was subjected to statistical analysis. The data was subjected to one-way ANOVA (Analysis of Variance) followed by Dunnett’s post test using Graph-pad prism Version 5.01 software.

## Results

### Phylogenetic position of source plant SMCTSR/CC/2019

**Fig. 1:**
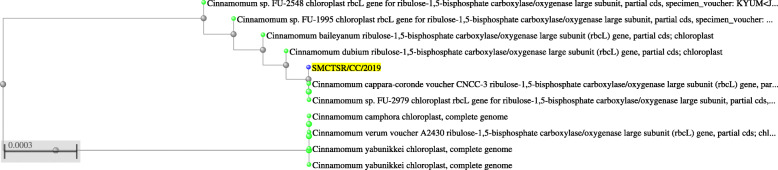
Phylogenetic position of *Cinnamomum cappara* SMCTSR/CC/2019 as per Blast analysis

The plant isolate SMCTSR/CC/2019 that could successfully exhibit protease mediated anthelmintic activity was identified by the employment of sequencing rbcL marker gene. From the phylogenetic tree (Fig. 1) created from Blast results, It was evident that the plant is *Cinnamomum cappara* belongs to family *Lauraceae* and order *Laurales*. Subsequently the Blast results and phylogenetic analysis of rbcL gene sequence stated that the source plant *Cinnamomum cappara* isolate SMCTSR/CC/2019 exhibited maximum similarity to *Cinnamomum cappara-coronde* whose sequence was deposited in NCBI under the accession number of MK243400.1 and JN407386.1 respectively.

### Proteolytic activity and effect of substrate concentration on the reaction velocity

**Fig. 2:**
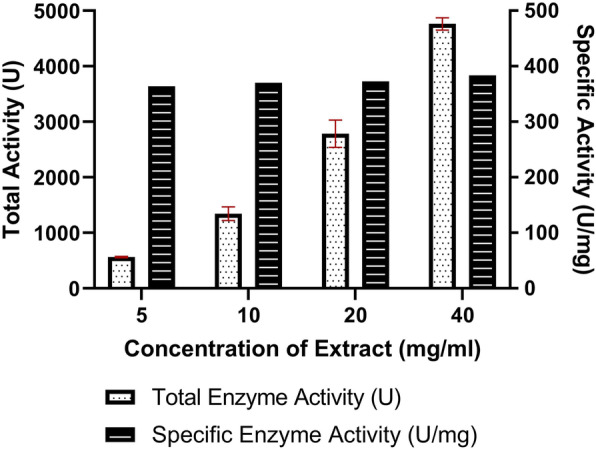
Total enzyme activity and specific enzyme activity exhibited by *Cinnamomum cappara* leaf extract

**Fig. 3:**
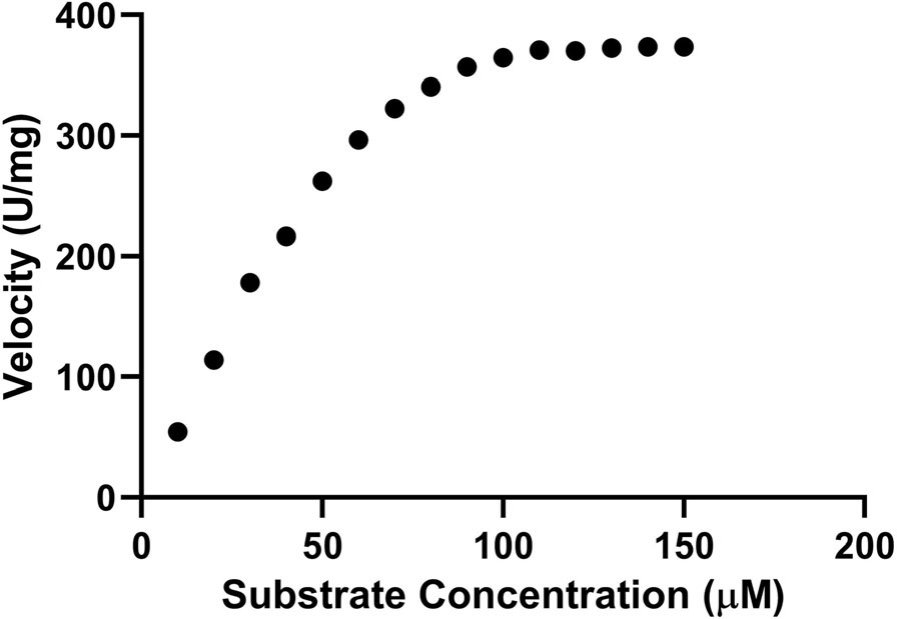
Effect of substrate concentration on proteolytic activity upon casein as substrate

**Fig. 4:**
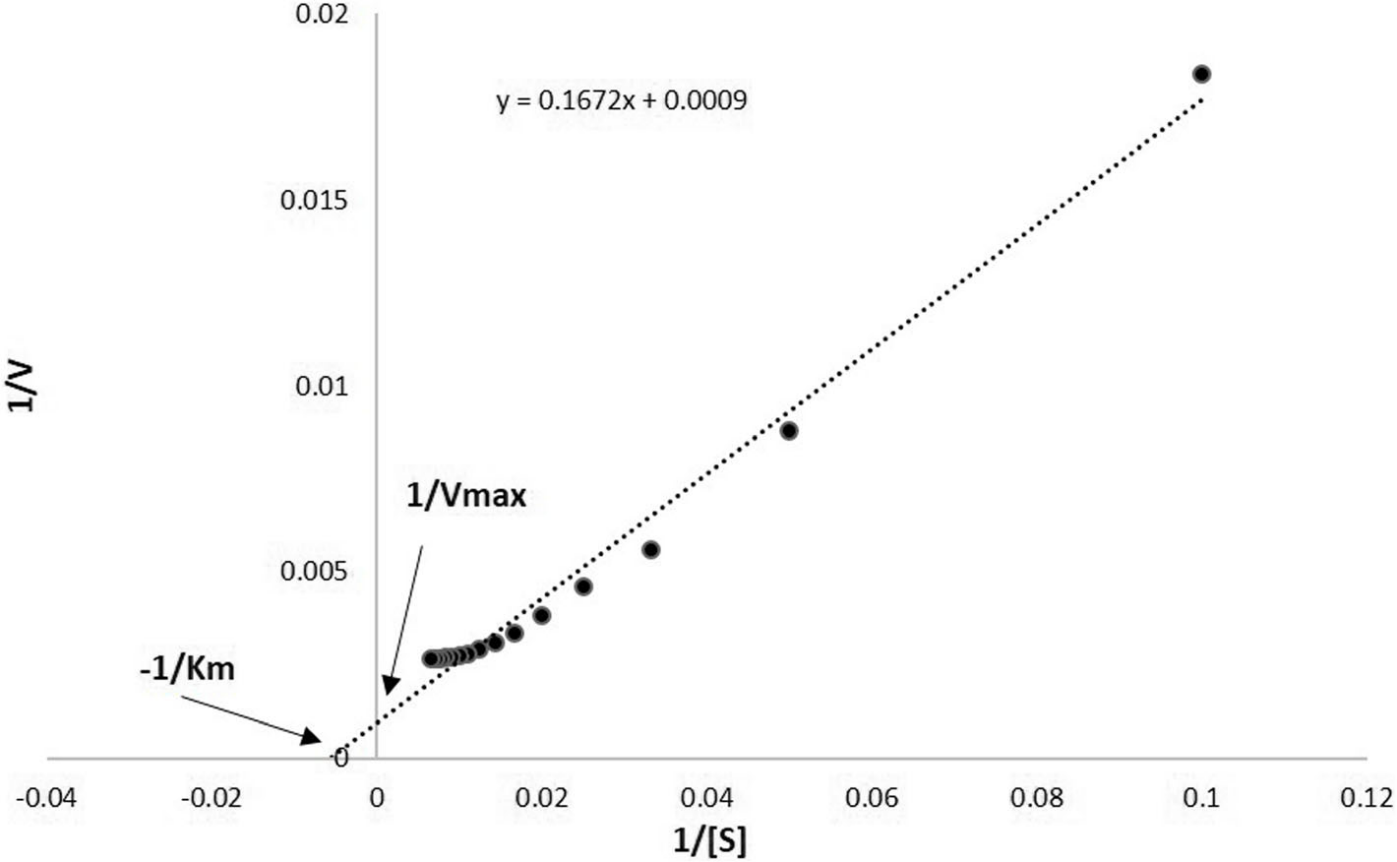
Lineweaver–Burk plot indicating Michaelis–Menten constant (Km), Maximum velocity (Vmax) and Substrate concentration [S]

*Cinnamomum cappara* leaf extract exhibited remarkable degree of Proteolytic activity where in the total enzymatic activity went up to 4763.66 ± 109.86 U in higher treated concentration that of 40 mg/ ml. The total enzymatic activity tends to be in correlation with that of concentration of plant extract wherein the Proteolytic action increased with increasing order of concentration (Fig. 2). It was interesting to understand that even though the total enzymatic activity was immensely influenced by the extract the specific enzyme action didn’t possess much variation (Table 1). From the studies conducted in different substrate concentration it was understood that the enzyme followed Michaelis–Menten kinetics. There was a typical hyperbolic relation with enzyme and increasing concentration of substrate (Fig. 3) wherein the enzyme saturation was visible in higher substrate concentration. From the Lineweaver–Burk plot (Fig. 4) the Km was found to be 185.77 µM for casein as substrate.

**Table 1:** Proteolytic activity of *Cinnamomum cappara leaf extract*

Concentration of Extract (mg/ml)	Total Enzyme Activity (U)	Total Protein (mg/ml)	Specific Enzyme Activity (U/mg)
5	565.33 ± 13.57	1.55 ± 0.22	363.94
10	1345.66 ± 124.27	3.63 ± 0.29	370.02
20	2784.66 ± 246.17	7.47 ± 0.05	372.61
40	4763.66 ± 109.86	12.42 ± 0.48	383.44

### Chemometric characteristics of bioactive fraction

**Fig. 5:**
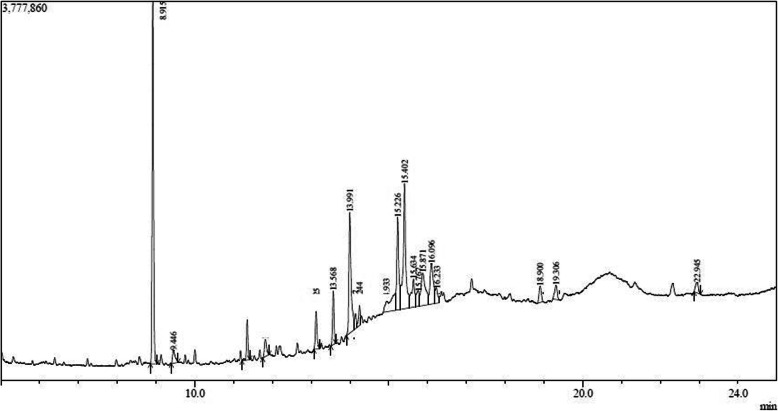
Chromatogram of bioactive fraction extracted from *Cinnamomum cappara* leaf

**Fig. 6:**
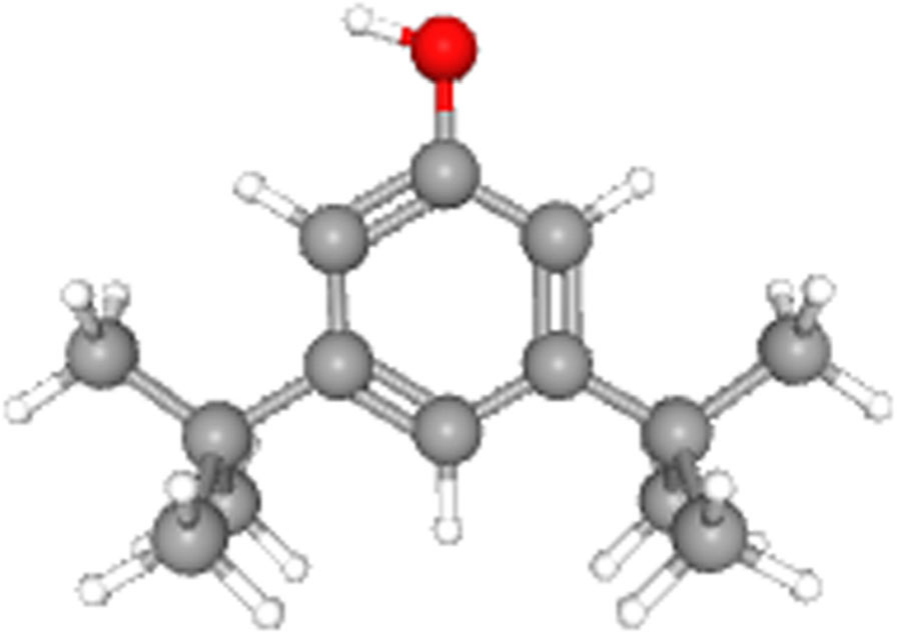
3D representation of Phenol, 3,5-di-tert-butyl

The bioactive fraction was subjected for gas chromatography and mass spectrometry subsequently to recognize the possible chemical composition of bioactive fractions. From the chromatogram obtained (Fig. 5) it was evident that the bioactive fraction of *Cinnamomum cappara* leaf extract comprises about seven major compounds and some trace components. Mass spectrometric analysis based on molecular mass suggested that the key component is Phenol, 3,5-di-tert-butyl (Fig. 6) which is an organic compound, with molecular formula C_14_H_22_O and molecular weight of 206.32 g/mol. This is colorless solid alkylated phenol and its derivatives are used industrially as UV stabilizers and antioxidants for hydrocarbon-based products. The peak was found in retention time 8.915.The identification was done by the employment of NIST library. Other Components those were found in the fraction was 9-Octadecenoic acid (Z)-, methyl ester which was present in retention time 15.402, 1-Pentadecanol (CAS) Pentadecanol which presented itself in retention time of 15.226. Other than these prominent compounds there were also some trace components in a negligible amount.

### Effect of ***Cinnamomum cappara*** leaf extract upon ***Pheretima posthuma***

**Fig. 7:**
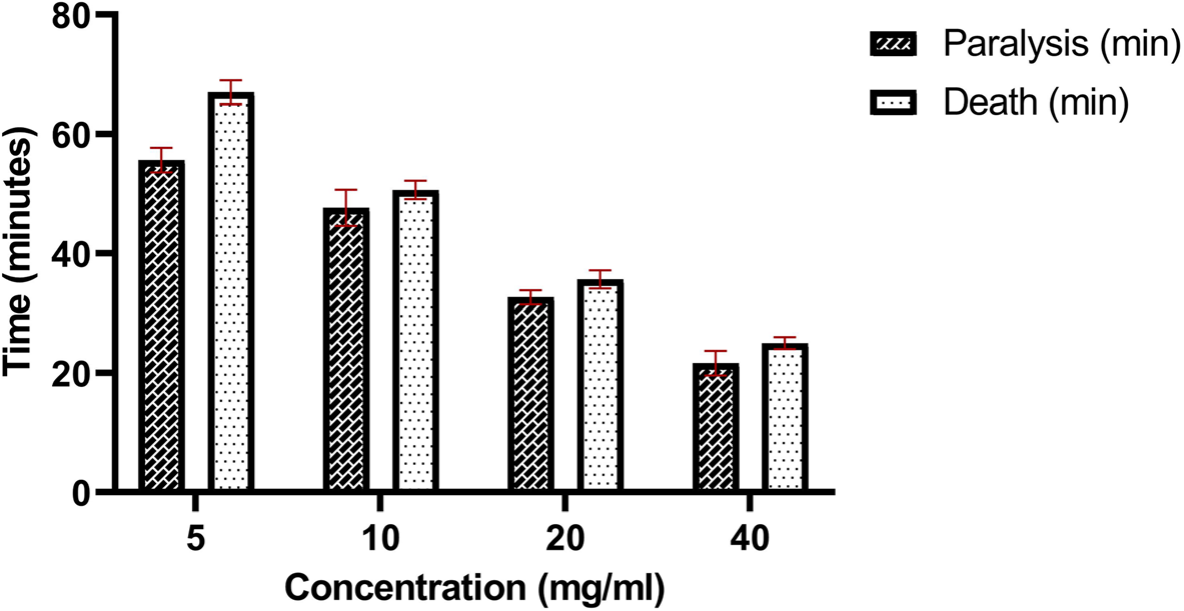
Effect of CCLE upon death and paralysis of *Pheretima posthuma*

**Fig. 8:**
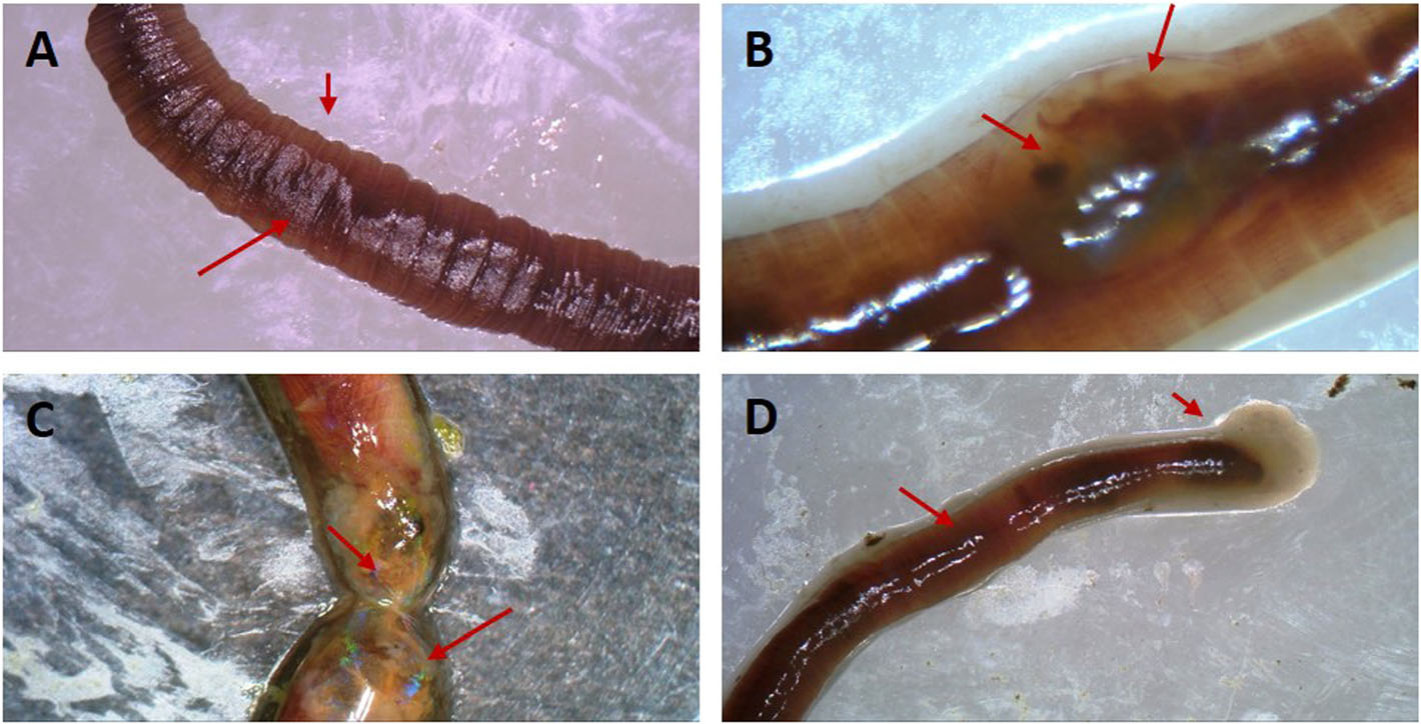
Anatomical and morphological aberrations caused by extract on *Pheretima posthuma*. **a**: Normal Anatomy; **b**: Treated samples displaying muscular contraction; **c**: Treated samples showing coelomic fluid leakage; **d**: Treated samples showing high degree of endothermal ablation

**Fig. 9:**
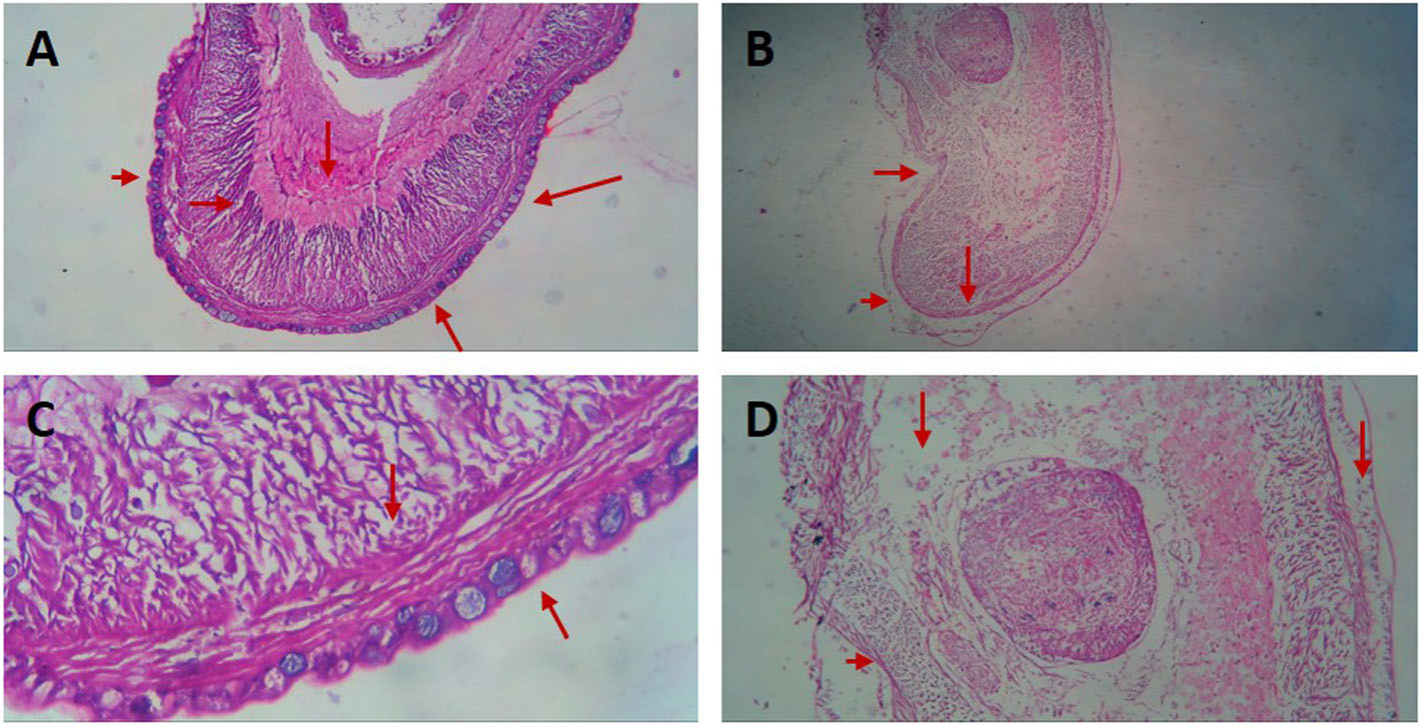
Histological analysis of effect of extract upon *Pheretima posthuma*. **a**: Photomicrograph showing histology of normal subject (10x). **b**: Histology of treated subject (10x). **c**: Photomicrograph showing histology of normal subject (40 x), showing compactly arranged muscular bundle with proper connective tissues. **d**: Histology of treated subject (40x) indicating the muscular degradation

The effect of *Cinnamomum cappara* leaf extract upon *Pheretima posthuma* suggested its potential anthelminthic property. The antagonizing activity was analyzed by subjecting *Pheretima posthuma* in varying concentrations (0 to 40 mg/ ml) of extract. It was observed that subjects exhibited diverse anatomical, morphological and behavioral changes in response to the treatment (Fig. 7). Escaping tendency was observed in all concentrations were in the higher concentration response was visible from 8th minute of administration wherein subjects remained peaceful in 5 mg/ml concentration up to 27th minute (Table 2). Non competence activity that of escaping moment accompanied by curling movement, wriggling movement etc. Higher degree of secretion of coelomic fluid was observed even in smaller concentrations. Subjects found to be getting paralyzed depending upon the concentration administrated. In higher concentration that of 40 mg/ml the paralysis was observed 21.66 ± 2.08 minutes after administration and found died at 25 ± 1 minute. In all the four different concentrations body elongation is the major morphological feature (Fig. 8). It was also evident that *Cinnamomum cappara* leaf extract could also affect the histological composition of *Pheretima posthuma.* Transverse sections of untreated earthworms indicated thick and intact epithelial layer, compact circular and longitudinal muscle layers, peritoneum and intestinal wall layers. Damaged cuticle and epithelial layers were found in the study animals in the presence of plant extract. Muscle layers were completely fused and vacuoles formed in between the longitudinal and circular muscles in this species. Thinning of epithelial layers was observed in clitellar region. Connective tissues were regularly degraded in that matter. Cellular debris was found in worms exposed to extract along with excessive mucilaginous secretion and slit degeneration of body wall layers (Fig. 9).

**Table 2:** Effect of extract in paralysis and death of Pheretima posthuma

Concentration(mg/ml)	Paralysis (min)	Death (min)
5	55.66 ± 2.08	67 ± 2
10	47.66 ± 3.05	50.66 ± 1.52
20	32.66 ± 1.15	35.66 ± 1.53
40	21.66 ± 2.08	25 ± 1

## Discussion

*Cinnamomum* (cinnamon) is a genus of the family *Lauraceae*, many of whose members are used as spices [18]. The genus comprises of small, evergreen trees and shrubs of about 10–15 m tall and are found in south-east Asia, China, and Australia as well as in Africa. The bark is widely used as spice, its leaves are ovate-oblong in shape, and exhibit a length of about 7–18 cm [19]. Flowers are arranged in panicles with a greenish color. The plant consists a purple colored fruit which is a 1 cm sized berry encompassing a single seed [20]. The genus *Cinnamomum* is considered to be highly medicinal and it has proven its properties such as free radicle scavenging, anticancer ablity, antimicrobial action, anti-hyperlipidemic etc. [21].

Due to the vast diversity and abundance of species range, marker based molecular identification is considered to be ideal for *Cinnamomum* Genus. Identification of plants by the employment of molecular markers give away the apprehension of errors that generally occur during identification based on morphological descriptions, therefore it is suggested to confirm the identity of plants by incorporating molecular markers as it offers high degree of species discrimination and fast results [22]. From the molecular sequencing we conducted we concluded that the plant belongs to *Cinnamomum cappara*. Similarly molecular characterization have been employed previously to confirm *Cinnamomum verum* Presl [23], trnL-trnF based identification of Cinnamomum spp [24] etc. We used rbcL (Ribulose bisphosphate carboxylase large chain) marker based identification as it is considered standard plant DNA barcoding markers due to their universality, relatively high overall sequence quality, low cost, and high discriminatory power between species[8, 25].

Proteolytic enzymes break the peptide bonds of proteins and they can be classified into acid, neutral, and alkaline proteases. Protease enzymes can be obtained from plants, animals, and microorganisms [26]. Our studies indicated that the plant extracts can be a virtuous proteolytic agents, similar suggestions have been previously made by Chinnadurai et al. (2018) where they studied the protease activity leaf extract of eighty medicinal plants. It is also perceived that the leaves of *Moringa oleifera* could successfully demonstrate peptide degrading activity [27]. From the studies we conducted it was evident that *Cinnamomum cappara* leaf extract possess itself with an immense ability of proteolytic action that can be exploited in pharmaceutics and industries. The total enzyme activity tends to increase directly proportional to the concentration, however the specific activity followed a standard range which could be due to the total protein content present in the extract as suggested by Feng et al., (2017) in their studies conducted by high-throughput estimation of specific activities of enzyme/mutants in cell lysates through immunoturbidimetric assay of proteins. It is also assumed that the antioxidant activities that is possessed by the extract might also have played a vital role in its anthelmintic activity [28]. The probable mechanism could be the activation of transcription factors that effect the natural physiological mechanism of subject [29].

Vast array of medicinal and industrial applications are displayed by phyto-proteases so far and some of them being the anti- Viral activity by the protease purified from *Azadirachta indica* and other medicinal plants [30], Cheese making ability by plant proteases extracted from *Cynara scolymus* L and *Silybum marianum* L. Gaertn [31]. However an important observation was made by Stepek et al., (2007) where they suggested that plant derived protease from kiwi fruit have remarkable Anthelmintic action against rodent stomach nematode, *Protospirura muricola.* This results were matching with the observations we made in our study where we observed protease enzyme mediated anthelminthic activity by *Cinnamomum cappara* leaf extract against *Pheretima posthuma.* The study organism resembles the similar physiological and anatomical structure with human intestinal parasites [32] therefore this observations shall be utilized against more parasites that cause Helminthiasis. Our studies suggested that proteolytic activity of plant extract could successfully cause degradation of muscle and hence death. Similarly various plant metabolites extracted from *Picria fel-terrae* Lour, *Linariantha bicolor, Lansium domesticum* have also reported their activity against Helminthiasis [33].

## Conclusions

*Cinnamomum cappara* leaf extract possessed high degree of protease intervened anthelmintic activity against *Pheretima posthuma.* The histological and anatomical studies suggested that the activity of extract was due to the degradation of muscular bundle of subject that resulted in the leakage of ceolomic fluid. The analysis upon enzymatic kinetics obtained by plotting Lineweaver–Burk plot suggested that the extract possess the Km 185.77 µM for casein as substrate and obeyed Michaelis–Menten kinetics with typical hyperbolic relation with enzyme and increasing concentration of substrate.

## Data Availability

All the data and materials will be made available upon request.
